# [Retracted] Resveratrol reduces acute lung injury in a LPS‑induced sepsis mouse model via activation of Sirt1

**DOI:** 10.3892/mmr.2023.13137

**Published:** 2023-11-28

**Authors:** Tongxun Li, Jinglan Zhang, Jiliang Feng, Qiang Li, Lisong Wu, Qing Ye, Jianping Sun, Yi Lin, Mengran Zhang, Rui Huang, Jun Cheng, Yongmei Cao, Guoan Xiang, Jinqian Zhang, Qinghua Wu

Mol Med Rep 7: 1889–1895, 2013; DOI: 10.3892/mmr.2013.1444

Following the publication of this paper, it was drawn to the Editor's attention by a concerned reader that the β-actin control western blotting data shown in Fig. 3D on p. 1893 were very similar to the contol data shown in Fig. 4A on p. 1894; furthermore, the data shown for the MMP-9 and the INOS protein bands in Fig. 4C were remarkably similar to the data shown for the IL-1β and IL-6 proteins, respectively, albeit the backgrounds surrounding the bands were different. Moreover, various of the western blotting data shown in these figures were strikingly similar to data that had already been published in different form in other articles written by (largely) different authors at different research institutes.

Owing to the fact that the contentious data in the above article had already been published prior to its submission to *Molecular Medicine Reports*, and due to the number of apparent duplications of strikingly similar data between Figs. 3 and 4, the Editor has decided that this paper should be retracted from the Journal. The authors were asked for an explanation to account for these concerns, but the Editorial Office did not receive a satisfactory reply. The Editor apologizes to the readership for any inconvenience caused.

